# Vitamin B1 Involved in *Dendrobium* Taiseed Tosnobile Extract Mediates Protection Against Cancer-Induced Muscle Wasting by Suppressing IL-6 Pathogenicity and Enhancing Myoblast Fusion

**DOI:** 10.3390/ijms262110704

**Published:** 2025-11-03

**Authors:** Chen-Chu Lin, Wan-Ting Liao, Tsung-Ying Yang, Jing-Hua Tsai, Yi-Ju Lee, Chi-Luan Wen, Shih-Lan Hsu, Chun-Chi Wu

**Affiliations:** 1 Institute of Medicine, Chung Shan Medical University, 110, Section 1, Jianguo N. Road, Taichung 402, Taiwan; 2 Chinese Medicine Department, Show Chwan Memorial Hospital, Changhua 508, Taiwan; 3 College of Medicine, National Chung Hsing University, Taichung 402, Taiwan; 4 Division of Chest Medicine, Department of Internal Medicine, Taichung Veterans General Hospital, Taichung 402, Taiwan; 5 Department of Life Sciences, National Chung Hsing University, Taichung 402, Taiwan; 6 Taiwan Seed Improvement and Propagation Station, Council of Agriculture, Propagation Technology Section, Taichung 426, Taiwan; 7 Department of Medical Research, Taichung Veterans General Hospital, Taichung 402, Taiwan; 8 Department of Medical Research, Chung Shan Medical University Hospital, 110, Section 1, Jianguo N. Road, Taichung 402, Taiwan; 9 Department of Health Industry Technology Management, Chung Shan Medical University, 110, Section 1, Jianguo N. Road, Taichung 402, Taiwan

**Keywords:** muscle wasting, *Dendrobium* Taiseed Tosnobile, IL-6, E-cadherin, MyHC, C2C12, multinucleation

## Abstract

In this report, we showed that oral administration of *Dendrobium* Taiseed Tosnobile (DTT, also known as Taiwan Emperor No.1) allowed Lewis Lung Carcinoma (LLC) tumor-bearing mice to maintain body weight and grip strength in a dose-dependent manner. Histological analysis showed that treatment with DTT water extract significantly reduced muscle fiber damage by inducing muscle regeneration and improved the cross-sectional area of the rectus femoris, soleus, and gastrocnemius of LLC tumor-bearing C57BL/6 female mice. Further studies revealed that DTT water extract also reduced the expression of inflammatory cytokines such as IL-6 and TNF-α, both in vitro and in vivo. Other analyses showed that DTT water extract promoted the differentiation of C2C12 myoblasts with or without IL-6 by maintaining Myosin Heavy Chain (MyHC) levels. This suggests that DTT water extract acts against muscle wasting via multiple mechanisms. Interestingly, vitamin B1 was identified as an ingredient in DTT water extract through an HPLC analysis. Vitamin B1 was shown to ameliorate IL-6 but not TNF-α generation in active THP-1 cells and protected C2C12 myotubes against IL-6. Further studies showed that DTT and vitamin B1 promoted the multi-nucleus fusion step of C2C12 differentiation by inducing E-cadherin-β-catenin expression with or without IL-6 treatment. In summary, DTT water extract protects muscle cells under cancer conditions through direct and indirect mechanisms, with vitamin B1 being a key functional ingredient that reduces IL-6 generation and aids muscle cell fusion against IL-6 treatment.

## 1. Introduction

Skeletal muscle is the most abundant tissue in healthy people (about 45 ± 5% of body mass) [1]. An individual skeletal muscle comprises bundles of muscle fibers enclosed by connective tissue sheathing [2]. The main components of each muscle fiber are striated bundles formed by the fusion of differentiated myocytes [3]. Myotubes are mature myofibrils containing multiple myofilaments, including actin, elastin, and MyHC [4]. Skeletal muscle fibers are generated by the repeated fusions of myocytes and myotubes during embryonic development. In adults, the fusion of myogenic satellite cells is essential to promote the growth and repair of myofibers after injury. This fusion of myocytes and myotubes is a complicated process that includes the remodeling of both the membrane and the cytoskeleton and dozens of proteins involved in this process, such as Interleukin-4 (IL-4), Vascular cell adhesion protein (V-CAM), myoferlin, MEK/ERK, and β-catenin [5,6,7,8,9]. Loss of function of these proteins affects the development or regeneration of muscle fibers. Skeletal muscles are essential for locomotion control, organ protection, maintaining posture, acting as a protein reservoir, and enabling daily physiological activities [10]. The optimal size and function of muscles depend on the balance between biosynthesis and the destruction of muscle cells [11].

Muscle wasting (or muscle atrophy), a condition of gradual loss of muscle mass and function, is particularly significant in older adults and patients with genetic defects, chronic diseases, or cancer cachexia [12,13]. Cachexia is the most critical comorbidity found in 40–80% of cancer patients, particularly at advanced stages of the disease, depending on the type and size of cancer [14]. Its high incidence of cachexia makes the management of cancer patients more complicated, leading to poor response to treatments, poor appetite, and decreased body strength, which generally results in a dramatic decrease in quality of life [15,16,17]. The consequence of cancer cachexia is medical futility and a poor survival rate, causing more than 25% of cancer deaths [18]. The pathogenetic characteristics of cancer cachexia include the loss of fat mass and skeletal muscle, often accompanied by anorexia; however, muscle wasting is considered the most critical trait since it is responsible for most of the symptoms associated with cancer cachexia and a poor prognostic factor of survival [19,20,21]. The underlying mechanisms of cancer cachexia are mainly involved in the up-regulation of inflammatory cytokines such as TNF-α and IL-6, which correlate with the imbalance between protein synthesis and protein degradation in muscle cells [22,23,24,25]. Some medications, including megestrol acetate or Adlumiz^®^ (a ghrelin activator), are appetite stimulants and have been reported to prevent weight loss and improve food intake in cancer cachexia patients. Despite validation, several adverse effects were reported [26,27,28,29]. Therefore, in addition to treatment with appetite stimulators, muscle wasting attenuation is considered an alternative strategy for anticachectic treatments.

Herb medicine, an integral part of Traditional Chinese Medicine (TCM), has attracted considerable attention due to its distinct efficacy in treating various diseases [30]. Dendrobii (Shi-Hu), a medicinal orchid (family: Orchidaceae) classified as a high-grade herb in “Shen Nong Ben Cao Jing,” has been widely used for general health improvement, such as antipyretic, ophthalmic, and tonic benefits [31,32,33]. While Herba dendrobii exhibits multiple health benefits and is a valuable herb on the market ($3000/kg), its yield is relatively low due to its rare natural distribution in the natural environment and slow growth rate. Herba dendrobii’s yield was relatively low [34,35]. To overcome this obstacle, a new species, *D.* Taiseed Tosnobile (DTT), was developed by crossbreeding two medicinal species, *D. tosaense* and *D. nobile*, by the Taiwan Seed Improvement and Propagation Station [36]. DTT is notable for its high production and easy cultivation compared to medicinal dendrobii [36]. Prior research showed DTT’s biological safety and immunomodulation functions [36,37]. However, it remains to be elucidated whether DTT exerts other health benefits. Based on previous studies and pharmacopeia records, we hypothesize that *Dendrobium*’s immunomodulatory capability may facilitate cancer patients’ rehabilitation. Therefore, this report investigates the effects of DTT water extract on cancer-induced muscle wasting and potential underlying mechanisms. Our results showed that administering DTT water extract effectively attenuated muscle wasting in LLC tumor-bearing mice. Further research revealed that DTT water extract protected myotubes from injury both directly and indirectly.

## 2. Results

### 2.1. DTT Water Extract Attenuates the Loss of Tumor-Free Body Mass in LLC Tumor-Bearing Mice

**Figure 1:**
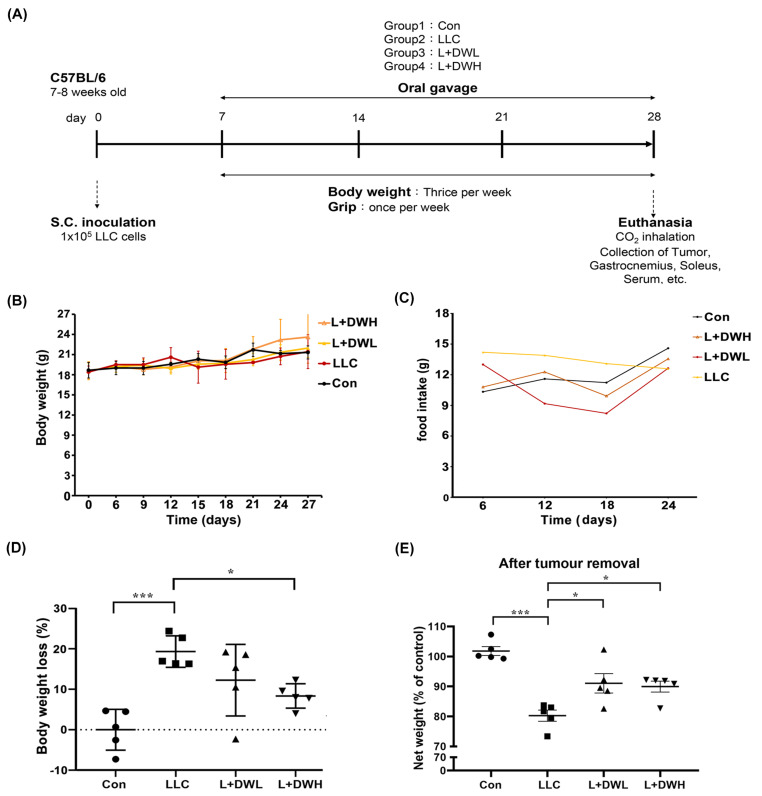
**DTT water extract reduces body weight loss in tumor-bearing mice with LLC.** (**A**) Treatment schedule for experimental animal study, Lewis lung cancer cells were implanted subcutaneously in the right flank of mice (1 × 10^5^/mouse) on day 0. DTT water extract (75 or 150 mg/kg/day) was started orally on day seven after tumor inoculation (tumor was apparent), while the tumor control group received an equal volume of water. From day seven, all groups of mice were subjected to a measurement of body weight week and grip strength once a week until day 28. The mice were then sacrificed, and related muscle tissues such as the left hind limb, gastrocnemius, and soleus of the right hind limb were collected for the indicated experiments. (**B**) Each group’s mean consecutive body weights were recorded every week until sacrifice. (**C**) The mean food intake of each group was recorded every day until sacrifice. (**D**) On the day of sacrifice, each group’s mean body weight (w/o tumor) was measured and compared with that of the control group as a related loss ratio, n = 5/group, *** *p* < 0.001, * *p* < 0.05. (**E**) The mean body weight without tumors on the day of the sacrifice of each group (n = 5/group) was presented, *** *p* < 0.001, * *p* < 0.05. Multiple group comparisons were achieved by one-way ANOVA with Tukey’s post hoc test.

To investigate whether DTT water extract mitigates cancer-associated muscle wasting, the LLC tumor-implanted mice model, as described in the Section 4, was utilized. Tumor-bearing mice began receiving DTT water extract orally every day starting on day 7 after LLC implantation and continued until day 28 (Figure 1A). At the experiment’s end, the net body mass of each mouse was determined after tumor removal. The results showed no significant differences in total weight or food intake between the experimental and control groups (Figure 1B,C). However, the mean net body mass of mice in LLC was nearly 20% lower compared to that of the control (Figure 1D). Interestingly, the administration of either low (75 mg/kg) or high (150 mg/kg) dose of DTT water extract effectively increased by 10–15% of net body mass compared to LLC tumor-bearing mice (Figure 1E) with no dose effects observed. This result suggests the possibility that the lowest dose of DTT water extract in this experiment was already beyond the minimal effective concentration needed to against weight loss in a tumor-bearing mouse. Furthermore, the results also showed that the net body mass of DTT water extract-treated mice on day 28 was, on average, 40% higher compared to their day 0 weight (Figure 1E). In contrast, control mice only increased by 25%. Since food intake was similar across all groups, this result suggests that the DTT water extract exhibits a better conversion ratio of food intake to body mass.

### 2.2. DTT Water Extract Sustains Muscle Mass and Grip Strength in LLC Tumor-Bearing Mice

**Figure 2:**
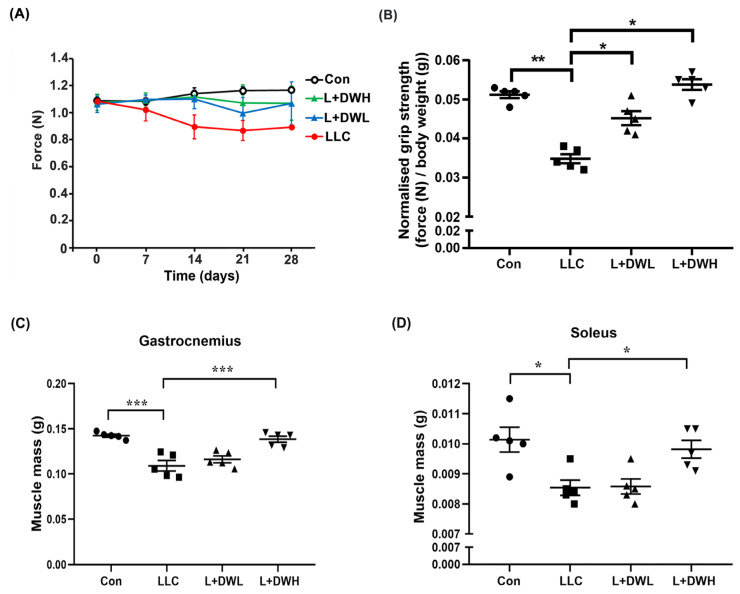
**DTT water extract retrieves the muscle functionality of LLC tumor-bearing mice.** (**A**) The grip strength of each group was measured once a week until day 28 and was shown. (**B**) On day 28, each group’s grip strength relative to body weights (without tumor) was recorded, and the average values were then calculated. Data are expressed as means ± SD, *** *p* < 0.001 compared to the control group, ** *p* < 0.01 compared to the tumor group. After the euthanasia of mice on day 28, the mass of (**C**) gastrocnemius and (**D**) soleus of each mouse from a different group was measured. n = 5/group, *** *p* < 0.001, * *p* < 0.05; one-way ANOVA achieved multiple group comparisons with Tukey’s post hoc test.

Our results showed that the DTT water extract significantly attenuated the body mass loss in LLC tumor-bearing mice. Since LLC tumors were known to prime continuous decline of muscle mass and eventually impede its functionality [38], we investigated whether DTT water extract prevented body mass loss by restoring muscle function and/or mass in LLC tumor-bearing mice. First, we analyzed the effects of DTT water extract on the grip strength of LLC tumor-bearing mice. The grip strengths of tumor-bearing mice decreased continuously as the experiment progressed and were reduced by 30–35% compared to the control on the day of sacrifice (Figure 2A). Concurrently, DTT administration promoted LLC tumor-bearing mice to maintain higher grip strength throughout the experiment. On day of sacrifice, the DTT water extract treated LLC mice increased about 20–25% grip strength compared to that of LLC tumor alone (Figure 2A). Furthermore, the measurement of F/W (force-over-body-weight) value on the day of sacrifice showed a reduction in F/W value in LLC tumor-bearing mice by 40% compared to the control (Figure 2B). Interestingly, administration of either low or high concentrations of DTT water extract recovered F/W values in mice carrying LLC tumors almost similar to that of the control (increasing by 60–65% compared to that of the LLC tumor alone) with no dosage effect observed (Figure 2B). This finding resembles the results seen for net body mass (Figure 1E). The results implied that DTT water-extract maintained muscle functionality by alleviating muscle atrophy in LLC tumor-bearing mice. To confirm this hypothesis, we investigated the variance in muscle mass across various experimental conditions. As excepted, remarkable mass reductions in the gastrocnemius and soleus muscle were observed by 22–31% in the presence of the LLC tumor compared to those of the control mice (Figure 2C,D), while the administration of high (150 mg/kg) but not the low (75 mg/kg) concentration of DTT water extract significantly increased the mass of the gastrocnemius and soleus by 20–25% in LLC tumor-bearing mice, almost the same weight as that of the control (Figure 2C,D). These findings collectively suggest that DTT water extract sustains muscle functionality in LLC tumor-bearing mice by maintaining muscle mass.

### 2.3. DTT Water Extract Suntains the Cross-Section Area of the Hindlimb in LLC Tumor-Bearing Mice

**Figure 3:**
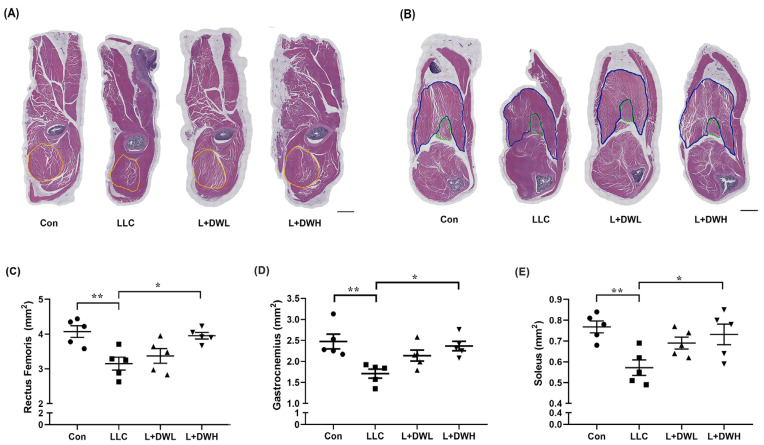
**DTT water extract ameliorates muscle CSA reduction in LLC tumor-bearing mice.** After euthanasia of the mice on day 28, the histopathological analysis of (**A**) rectus femoris, (**B**) soleus, and gastrocnemius of mice from control, LLC, LLC plus DTT water extract low dosage, and LLC plus DTT water extract high dosage were performed using H/E stain, scale bar = 1 mm. The cross-sectional area (CSA) of (**C**) Rectus femoris (yellow circle line), (**D**) soleus (green circle line), and (**E**) gastrocnemius (blue circle line) was calculated and quantified. n = 5/group, ** *p* < 0.005, * *p* < 0.05; multiple group comparisons were achieved by one-way ANOVA with Tukey’s post hoc.

To clarify how DTT water extract sustained muscle mass, we analyzed the effects of DTT water extract on the CSA (cross-sectional area) of the hindlimb muscles in tumor-bearing mice from LLC. Hematoxylin and Eosin (H&E) staining showed that the thigh and calf musculature were atrophic in LLC tumor mice, accompanied by reductions in perimysium, while the musculatures of LLC tumor-bearing mice treated with DTT water extract resembled those of the control group (Figure 3A,B and Figure S6). Further analysis revealed that the CSA of the rectus femoris (indicated by a yellow line) of tumor-bearing mice with LLC was smaller than that of the control group (reduction of 32–36%) (Figure 3A,C). The administration of high (150 mg/kg) but not low (75 mg/kg) concentrations of DTT water extract prevented this loss, increasing the rectus femoris CSA by 30–33% in LLC tumor-bearing mice and showed no significant difference compared to the control group (Figure 3C). Similar results were also observed in gastrocnemius (indicated by the blue line) and soleus (indicated by the green line), the CSA of these two musculatures reduced by the LLC tumor by 24–30% but the CSA was increased to levels almost similar to the control when the mice were treated with the high concentration of DTT water extract (Figure 3B,D,E). These results consistently suggest the requirement for a higher concentration of DTT water extract. This might be attributable to the relatively low concentration of active ingredients within the DTT water extract.

### 2.4. DTT Water Extract Alleviates Myofiber Atrophy Induced by LLC Tumor

**Figure 4:**
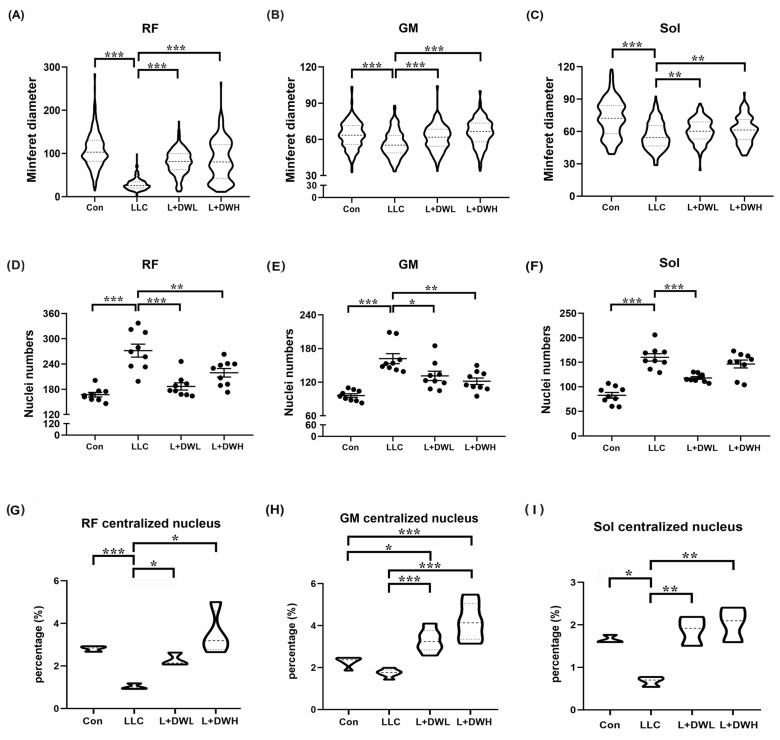
**DTT water extract maintained the architecture of the muscle fibers of LLC tumor-bearing mice.** The minferet diameter of the (**A**) rectus femoris, (**B**) gastrocnemius, and (**C**) soleus of each mouse was calculated and quantified. Four distinct fields of the nuclei of (**D**) rectus femoris, (**E**) gastrocnemius, and (**F**) soleus were calculated and quantified. The ratio of the centralized nucleus of (**G**) rectus femoris, (**H**) gastrocnemius, and (**I**) soleus was calculated and quantified. n = 5/group, *** *p* < 0.001, ** *p* < 0.005, * *p* < 0.05; one-way ANOVA achieved multiple group comparisons with Tukey’s post hoc test.

Next, we quantitatively measured the morphometric variations in the muscle fibers in mice under various experimental conditions. The initial analysis showed a significant reduction in the size of the perimysium and an irregular shape of the myofibers of rectus femoris, gastrocnemius, and soleus in LLC tumor-bearing mice compared to the control (Supplementary Figure S2A). The average minimum diameter (MinFeret) showed significant atrophy: an 80% reduction in the rectus femoris, a 19% reduction in the gastrocnemius, and a 28% reduction in the soleus compared to the control group. (Figure 4A–C). Furthermore, the administration of low and high concentrations of DTT water extract significantly increased the size of the myofibers of both the rectus femoris and soleus muscle by 65% and 26%, respectively, and almost fully recovered the size of the gastrocnemius muscle in LLC tumor-bearing mice (Figure 4A–C). The status of the nuclei of different musculatures was then investigated to confirm myofiber atrophy further. The number and aggregation of the nuclei were indeed increased in mice with LLC tumors compared to the control group (Supplementary Figure S2B). Quantitative results showed that the number of nuclei of the rectus femoris muscle in the LLC tumor-bearing increased by 85% compared to control group; similar patterns were observed in the gastrocnemius and soleus muscle, which increased by 72% and 98%, respectively (Figure 4D–F). Administration of both low- and high-dose DTT water extract in LLC tumor-bearing mice effectively reduced the abnormal nuclei number of the rectus femoris to the level of control, while only the high-dose DTT water extract was effective in the gastrocnemius muscle (reduced by 15–21% vs. LLC tumor alone). In contrast, the low dose of DTT water extract treatment only effectively reduced abnormal nuclei numbers in the soleus muscle (reduced by 20–25%) in LLC tumor-bearing mice (Figure 4D–F). Our results indicated that DTT water extract recovers muscle wasting by alleviating myofiber atrophy in LLC tumor-bearing mice. Furthermore, the variations in prevention among different types of muscle might be due to the multiple ingredients in the DTT water extract. The centralized nucleus is a hallmark of muscle regeneration [39], so this pattern was investigated to uncover underlying recovery mechanisms. The results showed the average ratios of the centralized nucleus of rectus femoris, gastrocnemius, and soleus were 2.8%, 2.3%, and 1.7%, respectively, in the control mice group. The ratio dropped to 1.0%, 1.7%, and 0.6% in the LLC-tumor mice group (Figure 4G–I and Figure S2C), indicating a reduction in muscle regeneration in a tumorous condition. Interestingly, the administration of both low and high doses of DTT water extract significantly increased the ratio of the centralized nucleus in rectus femoris, gastrocnemius, and soleus 2 to 3-fold across all three muscles in the LLC tumor group, resulting in ratios nearly equal to or higher than the control group (Figure 4G–I). Furthermore, this increase occurred in a dose-dependent manner. These data strongly suggested that the induction of muscle regeneration might be one pathway of DTT water extract against muscle wasting that is primed by tumorous conditions. Next, we investigated the architectures of muscle fibers in LLC tumor-bearing control mice with or without treatment with DTT water extract. The results showed that the area of the soleus/plantaris region of muscle cells decreased significantly in LLC tumor-bearing mice. Concurrently, the administration of DTT water extract recovered the architecture of muscle fibers (Supplementary Figure S3), which confirmed that the loss of muscle weight and area is attributable to reductions in muscle cell size/number.

### 2.5. DTT Water Extract Attenuates the Levels of Inflammatory Cytokines IL-6 and TNF-α Both In Vitro and In Vivo

**Figure 5:**
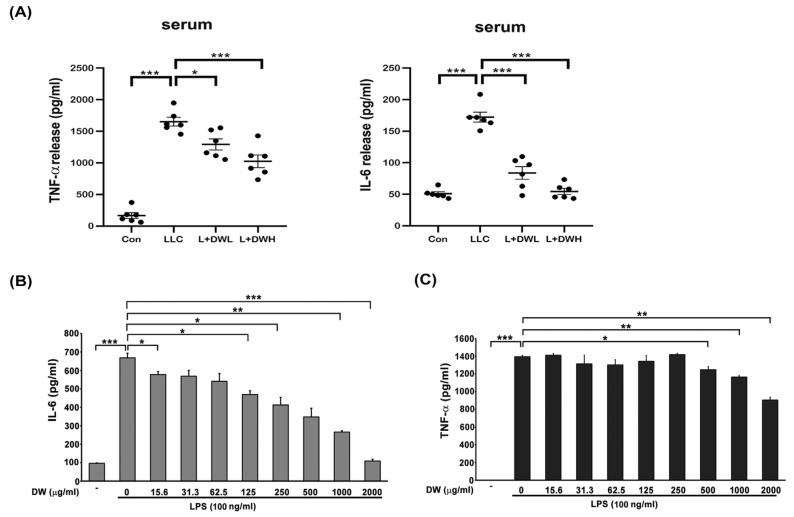
**DTT water extract in tumor-bearing mice inhibited the upregulation of serum IL-6 and TNF-α.** (**A**) After the euthanasia of mice on day 28, the serum of mice was collected, and the cytokines IL-6 and TNF-α concentrations were determined. (**B**) and (**C**) THP-1 cells were co-treated with LPS (100 ng/mL) with different doses of DTT water extract for eight hours, the medium was then collected and subjected to ELISA to reveal the levels of IL-6 or TNF-α. *** *p* < 0.001, ** *p* < 0.005, * *p* < 0.05; multiple group comparisons were achieved by one-way ANOVA with Tukey’s post hoc.

Both IL-6 and TNF-α were reported to be induced by tumors in both animal models and clinical investigations [40,41]. These two cytokines also showed the ability to induce muscle atrophy [24,25]. Therefore, we decided to examine the relationships between DTT water extract and IL-6 and TNF-α. The serum of LLC tumor-bearing mice exhibited a 2.5-fold increase in IL-6 compared to that of the control group, while administering a low dose of DTT water extract reduced serum IL-6 by 1.8-fold (relative to the control level). The high dose of DTT water extract nearly blocked IL-6 induction in LLC tumor-bearing mice (Figure 5A). However, the serum TNF-α increased 15–25 times by the LLC tumor compared to that of the control group; the treatment of low or high doses of DTT water extract reduced serum TNF-α specifically by 27% and 39%, respectively (Figure 5A). These in vivo results demonstrate an inverse relationship between DTT water extract administration and the circulating levels of the inflammatory cytokines IL-6 and TNF-α. To further dissect the causality and mechanism, we investigated the effect of DTT extract on cytokine generation in vitro. Activated THP-1 cells (Human Acute Monocytic Leukemia Cells) were pretreated with DTT water extract and subsequently challenged with LPS. The results showed that 125 μg/mL of the DTT water extract attenuated the expression of IL-6 induced by LPS by 18%. Furthermore, 500 μg/mL of DTT water extract reduced the level of IL-6 by 35% (Figure 5B), showing a dose-dependent effect. On the contrary, the inhibitory effects of DTT water extract on TNF-α were milder; 500 μg/mL of DTT water extract reduced TNF-α levels by only 9%. A significantly higher concentration of DTT was required to achieve a more notable reduction of 28% (Figure 5C). These findings indicate that DTT water extract inhibits the generation of both IL-6 and TNF-α, with a markedly more substantial effect on IL-6. This inhibitory action likely significantly attenuates tumor-induced muscle atrophy observed in vivo.

### 2.6. DTT Water Extract Protects C2C12 Differentiation Against IL-6

**Figure 6:**
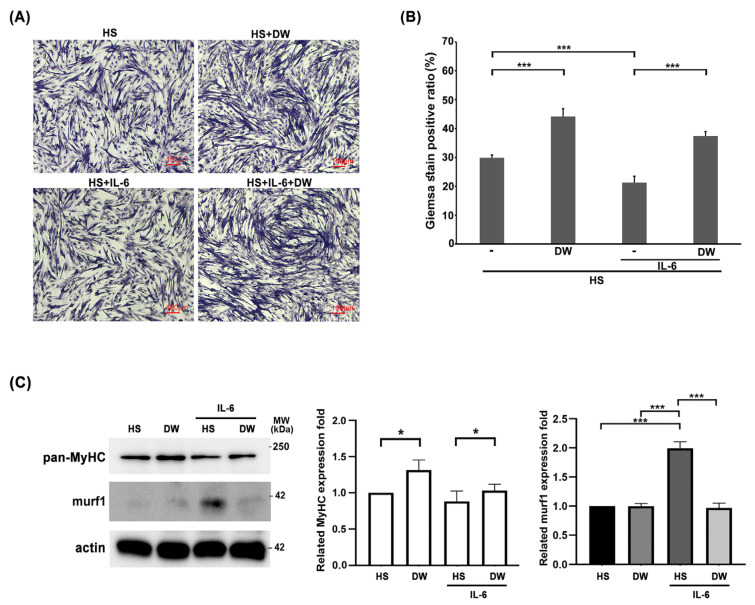
**The reductions in C2C12 myotubes by IL-6 were reversed in the presence of DTT water extract.** The C2C12 myoblasts were subjected to differentiation with 2% Horse Serum (HS) for 6 days with or without DTT water extract (800 μg/mL); the differentiated cells were then treated with 100 ng/mL of IL-6 for another 2 days. (**A**) Representative images were revealed using Giemsa stain. (**B**) GIEMSA positive signals were counted and calculated, *** *p* < 0.001. (**C**) The expressions of MyHC of different experimental C2C12 cells were revealed by Western blot using an anti-pan-MyHC or murf-1 antibody, respectively. The bottom chart shows the quantification results, *** *p* < 0.001, * *p* < 0.05.

We have shown that DTT water extract ameliorate muscle wasting by reducing the generation of IL-6 and TNF-α. Next, we focus on the direct effects of DTT in protecting muscle cells against IL-6. C2C12 myoblasts were differentiated into myocytes (one nucleus) or myotubes (≥two nuclei) with or without DTT water extract for six days, followed by IL-6 administration for another two days. The results showed an increasing ratio of differentiated C2C12 cells from 29% to 45% when treated with DTT water extract alone; Conversely, IL-6 treatment alone significantly reduced the differentiation ratio from 29% to 19% (Figure 6A). Pretreatment of DTT water extract maintained the C2C12 differentiation ratio at 41% even in the presence of IL-6 (Figure 6A,B). This finding strongly implies that DTT water directly protects muscle cells against IL-6. Western blot analysis showed the reduction in MyHC under IL-6 treatment (Figure 6C). DTT water extract significantly sustained the expressions of murf-1 and MyHC of C2C12 cells in the presence of IL-6. The molecular results are consistent with the results of Giemsa staining and support our earlier in vivo observation that DTT water extract improves muscle atrophy by stabilizing muscle fibers in the LLC mouse tumor model.

### 2.7. Vitamin B1 Is an Ingredient of DTT Water Extract Involved in Protecting Differentiated C2C12 Cells Against IL-6

**Figure 7:**
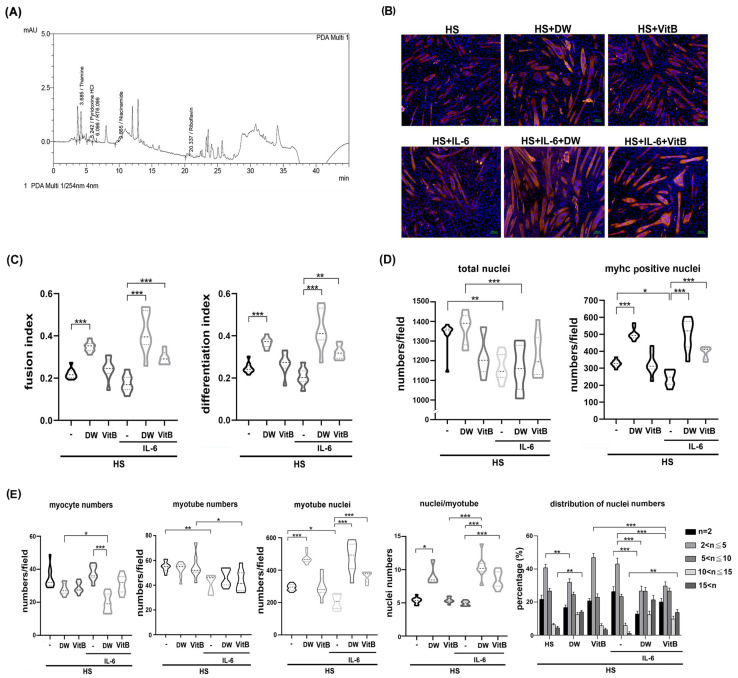
**Vitamin B1 is a potential functional ingredient of DTT water extract to promote the multinucleation of C2C12 myotubes reduced by IL-6.** (**A**) HPLC chromatogram revealed the different vitamin B derivatives of the DTT water extract. Peaks with an R time of 3.685, 5.242, 9.665, and 20.337 min corresponding to thiamine (vitamin B1), Pyrimidine HCl, niacinamide (vitamin B3), and Riboflavin (vitamin B2), respectively. (**B**) The C2C12 myoblast was subjected to differentiation with 2% horse serum for 6 days with or without the presence of DTT water extract (800 μg/mL) or vitamin B1 (5 μM); the differentiated myotube was then treated with 100 ng/mL of IL-6 for another 2 days. Representative images were revealed by immunofluorescence staining with anti-pan-MyHC antibody. The red indicates MyHC staining, and the blue represents the nucleus. (**C**)The fusion and differentiation index were calculated and quantified, respectively. *** *p* < 0.001, ** *p* < 0.005; multiple group comparisons were achieved by one-way ANOVA with Tukey’s post hoc test. (**D**) The total and MyHC-positive nucleus numbers were calculated and quantified, *** *p* < 0.001, ** *p* < 0.005, * *p* < 0.05, and multiple group comparisons were achieved by one-way ANOVA with Tukey’s post hoc. (**E**) The numbers of myocytes and myotubes, the total and mean nuclei numbers of myotubes, and the distributions of the nuclei numbers of C2C12 myotubes were calculated and quantified, *** *p* < 0.001, ** *p* < 0.005, * *p* < 0.05. Two-way ANOVA achieved multiple group comparisons with Tukey’s post hoc.

To dissect the possible ingredients of DTT responsible for the protective effects of muscle cells against IL-6, we performed an HPLC assay to identify potential constituents of DTT water extract as described in the Section 4. Various vitamin B derivatives, namely thiamin (vitamin B1), pyridoxine (vitamin B6), niacinamide (vitamin B3), and Riboflavin (vitamin B2), were detected at retention times of 3.685 min, 5.242 min, 9.665 min, and 20.337 min, respectively (Figure 7A) (Supplementary Figure S7). Thiamin (Vitamin B1) accounted for the highest percentage among these Vitamin B derivatives. Given this finding, we investigated the inhibitory effects of vitamin B in regulating pro-inflammatory cytokines such as IL-6 and TNF-α of active THP-1cells. The results showed that administration of vitamin B1 effectively reduced the generation of IL-6 but not TNF-α in active THP-1 cells (Supplementary Figure S4), indicating that vitamin B1 is involved in the partial reduction in pro-inflammatory cytokines. Next, we analyzed the effects of vitamin B1 on the protection of differentiated C2C12 cells against IL-6. To further clarify the differential effects of DTT water extract or vitamin B1 in regulating C2C12 differentiation, the immunofluorescence analysis was performed using an anti-pan-MyHC antibody. The results showed that both the fusion index (the numbers of nuclei of cells whose nucleus numbers are larger than one with MyHC positive were divided by total nuclei) and the differentiation index (the numbers of nuclei of cells with MyHC positive were divided by total nuclei) of C2C12 cells treated with DTT water extract were higher than untreated (increased by 15–20%, Figure 7B,C), while vitamin B1 treatment showed a similar trend to DTT water extract; however, the difference did not reach a statistically significance. IL-6 treatment reduced both fusion and differentiation index compared to untreated cells. Interestingly, the pre-addition of DTT water extract showed a nearly 2-fold increase in both fusion and differentiation index of C2C12 cells treated with IL-6 alone. Vitamin B1 exhibited a similar but minor induction of the fusion and differentiation index with that of IL-6 treatment alone (an increase of 90–95%, Figure 7B,C). The total number of nuclei and the number of MyHC-positive cells were significantly lower in IL-6-treated cells than in untreated cells. This suggests that the IL-6-induced loss of cells and myotubes might explain why neither fusion nor differentiation index showed a clear difference between the differentiated C2C12 cells treated or untreated with IL-6 (Figure 7B,D). The ratio of MyHC-positive cells in C2C12 differentiated cells treated with DTT water extract was significantly higher than that in untreated cells (increasing by 70–80%). Total nuclei numbers, however, were not affected, while vitamin B1 treatment showed a similar increase compared to untreated cells (Figure 7D). Interestingly, DTT water extract pretreatment maintained a high ratio of MyHC positive cells in the presence of IL-6 (increased by 90–110% compared to IL-6 alone); Vitamin B1 pretreatment also exhibited a protective effect, showing a higher ratio of MyHC-positive cells compared to IL-6 alone (increased by 55–65%). These results indicated that vitamin B1 is an ingredient in DTT water extract to maintain differentiated C2C12 myotubes against IL-6. Further results showed that the number of single-nucleated, MyHC-positive myocytes remained similar among untreated, DTT water extract-treated, and vitamin B1-treated C2C12 cells. Furthermore, the coexistence of DTT water extract and IL-6 significantly reduced the ratio of myocytes compared to that of untreated, DTT water extract, or IL-6 alone, while vitamin B1 pretreatment exhibited a similar but non- significant reduction in myocyte numbers in the presence of IL-6 (Figure 7E). Unexpectedly, the numbers of myotubes of DTT water extract or vitamin B1 treatment were lower than those of the untreated ones (reduced by 12–17% vs. untreated, Figure 7E) and even lower in the presence of IL-6 (reduced by 22–35% vs. untreated; reduced by 10–15% vs. IL-6 alone, Figure 7E) (Supplementary Figure S7). Analysis of multinucleated, MyHC-positive myotubes (nucleus number/cell > one) revealed that DTT water extract but not vitamin B1 treatment significantly increased the total number of nuclei within these myotubes compared to untreated cells (increased by 75–92% vs. untreated, Figure 7E). Treatment with IL-6 significantly reduced nuclei numbers per myotube by 20–30% compared to those of untreated ones, surprisingly. DTT water extract pretreatment followed by IL-6 administration caused a robust increase in nuclei numbers per myotube by 172–221% compared to IL-6 alone (Figure 7E and Figure S7). Similar trends were also observed when pretreatment with vitamin B1 was followed by IL-6 (increased by 145–195%, Figure 7E, bottom left panel and Supplementary Figure S7). The increase in nuclei count concurrent with a reduction in myotubes following treatment with either DTT water extract or vitamin B1 raises the possibility that these agents promote the multinucleation process in C2C12 myotubes, irrespective of IL-6 co-treatment. The numbers of nuclei per myotube were then calculated to validate the possible effects. As expected, the average number of nuclei in C2C12 myotubes treated with DTT water extract increased by 55–110% compared to untreated (Figure 7E, bottom left panel). Furthermore, the co-presence of IL-6 and DTT water extract induced higher average nuclei numbers of C2C12 myotubes up to 200–230% compared to untreated ones. A smaller induction was also observed as pretreatment of vitamin B1 followed by IL-6 (increased by 35–40% compared to IL-6 alone, Figure 7E, bottom left panel). In the analysis of the nuclei number distribution of the myotube, the highest distribution of nuclei numbers of untreated C2C12 myotubes was 2–5 per myotube (nearly 62%), and the nuclei number ratio greater than 10 per myotube was approximately 8–10%; however, the ratio of large myotubes (>10 nuclei) increased significantly by up to 35–38% while the nuclei number ratio at 2–5 was reduced by 15% with the treatment of DTT water extract (Figure 7E, bottom right panel and Supplementary Figure S7). On the other hand, vitamin B1 or IL-6 treatment did not show any significant differences in the distributions of nuclei numbers per myotube compared to untreated ones (Figure 7E, bottom left panel). IL-6 treatment reduced the ratio of nuclei numbers more than 10 per myotube by 51–62% (vs. untreated), while the presence of DTT water extract dramatically induced the ratio of high nucleus numbers (>10) per myotube from 4–7% to 22–27%. In contrast, the number of nuclei in 2–5 per myotube in the coexistence of IL-6 and DTT water extract was reduced from 60% to 38% compared to IL-6 alone (Figure 7E, bottom right panel). Furthermore, pretreatment with vitamin B1 also showed a similar but smaller change in the high numbers of nuclei (>10) per myotube in the presence of IL-6 compared to treatment with IL-6 alone (23–27% vs. 4–7%, Figure 7E, bottom right panel and Supplementary Figure S7). These combined results indicate that DTT water extract promotes the mid-to-late stages of myoblast differentiation, specifically enhancing the multinucleation of large myotubes into myofilament by encouraging the fusion of smaller myotubes or myocytes, rather than solely promoting early differentiation. In addition, the pathological effects of IL-6 on C2C12 differentiation would be reversed in the presence of DTT water extract, which could explain the protective effects of DTT water extract in cancer-induced muscle wasting. Regarding vitamin B1, despite treatment alone showing no significant effects as DTT water extract in the presence of IL-6, vitamin B1 exhibited an induction capacity of the fusion index, differentiation index, and multinucleation of myotubes. These results suggested that vitamin B1 might act alone or cooperate with other ingredients of DTT water extract to sustain the differentiation process, such as multinucleation of C2C12 in different circumstances.

### 2.8. E-Cadherin Is Required for Multinucleation of C2C12 Myotubes Induced by DTT Water Extract or Vitamin B1

**Figure 8:**
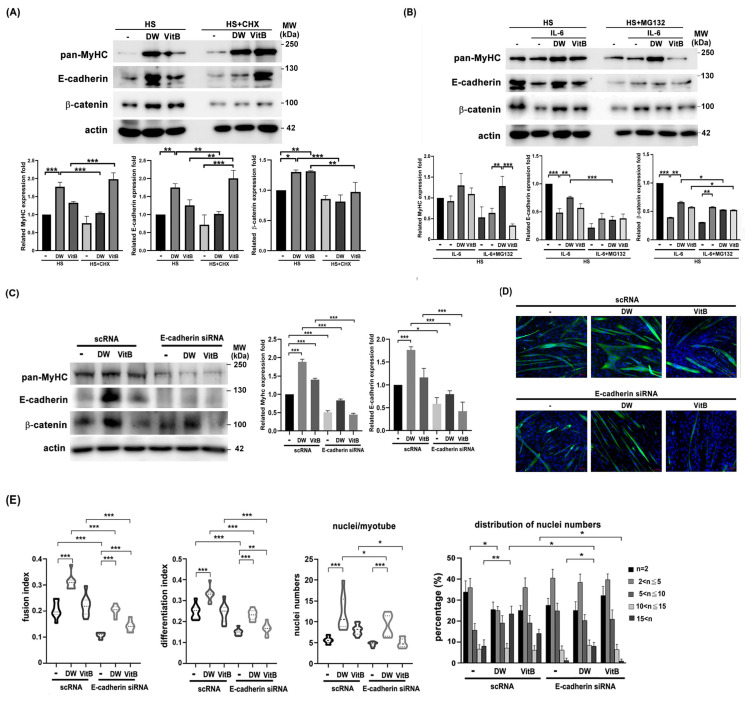
**E-cadherin/β-catenin is accompanied by multinucleations of C2C12 myotubes induced by DTT water extract or vitamin B1.** (**A**) The C2C12 myoblasts were differentiated for 6 days with or without DTT water extract (800 μg/mL) or vitamin B1 (5 μM). Two hours before harvesting, cells were treated with cycloheximide, followed by collection, extraction, and subjected to Western blotting using anti-pan-MyHC, E-cadherin, β-catenin, or β-actin antibodies, respectively. The expression of β-actin served as an internal control. Quantification results of Western blots are shown in the bottom chart, *** *p* < 0.001, ** *p* < 0.005, * *p* < 0.05 (**B**) The C2C12 myoblast was subjected to differentiation for 6 days with or without the presence of DTT water extract (800 μg/mL) or vitamin B1 (5 μM); the differentiated myotube was then added with 100 ng/mL of IL-6 for another 2 days. Cells were treated with MG132 two hours before harvesting, and Western blots were performed. Quantification results of Western blots are shown in the bottom chart, *** *p* < 0.001, ** *p* < 0.005, * *p* < 0.05. The scRNA or E-cadherin siRNA-transfected C2C12 cells were subjected to differentiation for 6 days with or without DTT water extract (800 μg/mL) or vitamin B1 (5 μM). The cells were then collected and subjected to (**C**) a western assay using an anti-E-cadherin antibody; the quantification results of the Western blot are shown as the bottom chart, *** *p* < 0.001, * *p* < 0.05, or (**D**) immunofluorescence using an anti-pan-MyHC antibody. (**E**) The fusion index, the number of differentiation index, nuclei/myotube, and nuclei distribution ratio from C2C12 cells of different groups were counted and calculated. *** *p* < 0.001, ** *p* < 0.005, * *p* < 0.05; two-way ANOVA achieved multiple group comparisons with Tukey’s post hoc test.

Next, we performed a Western blot analysis to identify molecules affected by DTT water extract during C2C12 differentiation, both with and without IL-6 co-treatment. Results showed that DTT water extract significantly upregulated MyHC protein levels by 1.5- to 2.4-fold compared to the untreated control. A similar trend was observed with vitamin B1, which increased MyHC by 45–55% (Figure 8A). Furthermore, DTT water extract markedly induced the expression of adhesion proteins, elevating E-cadherin by 3- to 4.5-fold and β-catenin to a comparable extent. In contrast, vitamin B1 only increased E-cadherin levels by 0.7- to 1.5-fold over the control (Figure 8A). The addition of CHX (cycloheximide) reduced the levels of E-cadherin and β-catenin more significantly than MyHC in the presence of DTT water extract (Figure 8A). These results implied that DTT water extract might induce these molecules via de novo protein synthesis. Furthermore, IL-6 treatment reduced MyHC, E-cadherin, and β-catenin levels (35%, 80%, and 60% vs. untreated); Pretreatment of DTT water extract significantly maintained the levels of all three proteins (Figure 8B). However, pretreatment with vitamin B1 maintained MyHC, E-cadherin, and β-catenin levels at a minor trend (Figure 8B), indicating that some other ingredients of DTT water extract could also participate in up-regulation of these molecules in the presence of both DTT water extract and IL-6. It should be noted that the addition of MG132 only increased the level of β-catenin but not MyHC or E-cadherin (Figure 8B), suggesting that IL-6 reduced the levels of different proteins through different mechanisms. To elucidate the role of E-cadherin in C2C12 myogenesis, myoblasts were transfected twice with scramble RNA or E-cadherin siRNA, followed by myogenesis with or without DTT water extract or Vitamin B1. E-cadherin knockdown reduced β-catenin levels and impaired myotube formation, regardless of DTT water extract or vitamin B1 treatment (Figure 8C,D). Quantitative analysis further revealed that E-cadherin silencing markedly decreased both the fusion index and differentiation index to 9–13% of control levels (Figure 8E). Treatment with DTT water extract increased the fusion index to 33–38% in scramble RNA-transfected cells (scRNA + DW vs. scRNA control). However, this induction was attenuated to 17–20% upon E-cadherin knockdown (E-cadherin siRNA + DW vs. scRNA + DW), indicating that E-cadherin is essential for the pro-fusogenic effect of DTT water extract (Figure 8E). A similar trend was observed with vitamin B1 treatment, where E-cadherin depletion also reduced both the fusion and differentiation indices (Figure 8E). Although not statistically significant, E-cadherin knockdown also led to a reduction in both the average number of nuclei per myotube and the extent of multinucleation in C2C12 cells (Figure 8E). Consistent with prior findings, DTT water extract treatment increased the mean number of nuclei per myotube from 5–7 to 10–20 (scRNA + DW vs. scRNA; Figure 8E, bottom left). Additionally, the proportion of myotubes containing more than 15 nuclei rose from 7% to 26%, while those with 2–5 nuclei decreased from 38% to 23% (scRNA + DW vs. scRNA; Figure 8E, bottom right). Notably, E-cadherin knockdown reduced the proportion of myotubes with > 15 nuclei from 26% to 9% in DTT water extract-treated cultures (scRNA + DW vs. E-cadherin siRNA + DW; Figure 8E, bottom right). Nevertheless, DTT water extract still elevated the proportion of highly multinucleated myotubes (>15 nuclei) from 3% to 9% even under E-cadherin-depleted conditions (E-cadherin siRNA vs. E-cadherin siRNA + DW; Figure 8D,E), suggesting the involvement of additional molecules in DTT extract-induced multinucleation. Similarly, in vitamin B1-treated cells, E-cadherin knockdown reduced the proportion of myotubes with >15 nuclei from 15% to 3% (scRNA + VitB vs. E-cadherin siRNA + VitB; Figure 8E, bottom right), implying that vitamin B1 contributes partially to the multinucleation induced by DTT water extract via E-cadherin upregulation. In summary, these findings identify E-cadherin as a novel promoter of myotube multinucleation induced by DTT water extract. They underscore its role in counteracting IL-6-mediated muscle fiber disruption in a cachexia model.

## 3. Discussion

Skeletal muscles are essential for both metabolic health and daily activities. Loss of muscle mass or function often impairs mobility in older adults or patients with chronic diseases such as cancer. Preserving muscle function is key to maintaining quality of life and extending survival in elderly individuals or patients. Therefore, the search for effective and low-toxicity compounds to ameliorate muscle wasting induced by diseases has become a common goal in contemporary cachexia therapy or prevention. This report demonstrates that DTT water-extract administration preserved grip strength and muscle mass in LLC tumor-bearing mice. Furthermore, we showed that the reduction in the cross-sectional area, abnormal aggregation of nuclei (caused by muscle fiber atrophy), and decreased central nucleation (a hallmark of muscle regeneration), as well as reduced MyHC expression, were all ameliorated by treatment with the DTT water extract. Elevated serum IL-6 and TNF-α, both known to affect muscle mass and functions, were also reduced following DTT water extract administration. Furthermore, the in vitro assay showed that DTT water extract promoted the differentiation of C2C12 myotubes in the presence of IL-6. These results implied a dual role for DTT water extract in promoting myoblast differentiation and stabilizing myotubes. We then identified vitamin B derivatives as ingredients in the DTT water extract. Interestingly, our results indicated that vitamin B1 contributed to stabilizing differentiating C2C12 myotubes in the presence of IL-6, rather than promoting C2C12 cell differentiation. This suggests that vitamin B1 may act alone or synergize with other compounds of the DTT water extract to maintain differentiation of C2C12 in different conditions.

Dendrobii (*Dendrobium*), one of the largest genera in the Orchidaceae family, is known for its numerous medicinal effects, including anti-diabetic, anti-angiogenic, anti-inflammatory, anti-fungal, anti-bacterial, and anti-tumor activities [42,43,44,45,46]. Most of the effects are contributed by *Dendrobium* polysaccharides, alkaloids, or organic soluble compounds [35,47,48]. For example, gigantol from *D. Draconis* was shown to attenuate the stemness of lung cancer cells [49]; denbinobin from *D. nobile* can block the growth or motility of lung, ovarian, prostate, and colon cancer cells [31,50,51,52,53]; glucomannan from *D. officinale* regulates the production of IL-1β and TNF-α in THP-1 cells [54]; and phenanthrenes from *D. nobile* suppress LPS-induced NO generation in RAW 264.7 macrophages [55]. However, the potential of *Dendrobium* to ameliorate muscle wasting induced by cachexia or aging has not yet been reported. The present study is the first to demonstrate that DTT, a novel and endemic *Orchidaceae* species from Taiwan, exerts anti-muscle-wasting effects in tumor-bearing mice. Furthermore, we are the first to report that vitamin B1, one of the water-soluble components of DTT, helps protect differentiated C2C12 myocytes and myotubes against IL-6-induced damage. DTT is a new hybrid orchid bred by the Taiwan Seed Improvement and Propagation Station, and previous studies have reported its immunomodulatory properties [36,37]. Our findings expand DTT’s potential applications to preserve muscle mass and functions under pathological conditions. Given that DTT does not exert acute or subchronic toxicity and genotoxic effects [39], coupled with its high yield and easy cultivation, it represents a highly valuable medicinal herb with promising prospects in the health food market.

Several studies reported the effects of tumors in eliciting muscle wasting in different animal models. For example, reduced mass of the extensor digitorum longus, gastrocnemius, quadriceps, plantaris, tibialis anterior, and triceps muscles, as well as the soleus, along with diminished grip strength, have been observed in mice bearing tumor xenografts [56,57,58,59,60,61]. Consistent with previous reports, our results showed a similar reduction ratio of the mass of the rectus femoris, gastrocnemius, and soleus muscles in a tumor-bearing mouse model (Figure 3). Additionally, a reduction in the cross-sectional area and the diameters of the rectus femoris, gastrocnemius and soleus muscles along with multinucleation of myocytes, were also observed (Figure 4), indicating extensive muscle wasting induced by LLC tumor xenograft. Treatment with DTT water extract recovered the grip strength, fiber diameter, and nuclear number in the rectus femoris, gastrocnemius, and soleus muscles in a dose-dependent manner in tumor-bearing mice compared to control mice (Figure 3 and Figure 4). However, these muscles’ masses and cross-sectional areas were sustained only by the high dose of DTT water extract (Figure 3 and Figure 4). This raises the possibility that, although skeletal muscle’s physiological characteristics and performance are highly correlated in tumor-bearing mice, grip strength may depend more on myofibers’ diameter and nuclear patterning.

Zhang et al. reported that Baoyuan Jiedu Decoction ameliorated muscle wasting along with tumor inhibition, suggesting an indirect pathway for restoring muscle function in tumor-bearing mice [62]. Our results did not show statistically significant tumor inhibition when treated with DTT water extract (Supplementary Figure S5); however, substantial reductions in serum IL-6 and TNF-α, both of which are highly correlated with muscle atrophy in cancer cachexia models [63,64], were observed in the presence of DTT water extract in tumor-bearing mice. The in vitro results indicated that DTT water extract reduced the secretion of both IL-6 and TNF-α from activated macrophages (Figure 5). Previous studies and our results demonstrate that these inflammatory cytokines are essential in the cachexia process, and blocking them with DTT water extract could help maintain muscle mass and function even when tumors persist. We also showed that DTT water extract directly protected C2C12 myotubes under high-dose IL-6 treatment (Figure 6 and Figure 7). It should be noted that the role of IL-6 in the myogenesis of C2C12 is complicated; Steyn et al. showed that IL-6 promoted C2C12 cell differentiation, while Pelosi et al. showed that IL-6 impaired myogenesis of C2C12 [24,65]. This discrepancy is likely due to differences in the concentration and timing of IL-6 administration. Generally, adding a lower dosage of IL-6 (<100 ng/mL) at the start of differentiation promotes the differentiation index of C2C12 cells; however, adding IL-6 to already differentiated C2C12 myotubes causes myotube shrinkage. Indeed, our unpublished results showed that adding both DTT water extract and a low dose of IL-6 at the beginning of differentiation resulted in a higher differentiation index of C2C12 cells compared to treatment with the water extract alone, suggesting a dual role of IL-6 in promoting myoblast differentiation and altering differentiated myotubes. A similar pattern was observed for TNF-α, where pretreatment with DTT water extract ameliorated its detrimental effects. Our results demonstrated that DTT water extract protects muscle cells by reducing inflammatory cytokine levels and stabilizing myotubes in the presence of both IL-6 and TNF-α. It should be noted that cytokine storms, characterized by a surge in IL-6 and TNF-α, lead to muscle catabolism, impaired anabolism, and skeletal muscle atrophy in many other conditions, such as burns [66] and sepsis [67,68], indicating that DTT could also be relevant for muscle wasting beyond that associated with cancer. Therefore, further studies are warranted to investigate the effects of DTT on skeletal muscle wasting in various contexts.

DTT was crossbred by *D. nobile* and *D. tosaense* and was characterized by high yield and ease of cultivation. Yang et al. reported the immunostimulatory effect of the polysaccharide fraction from DTT water extract, which contrasts with our findings (Figure 5). This discrepancy might be due to differences in the amount and purity of polysaccharides used in different experiments. The physiological effects of DTT water extract and its corresponding active ingredients have not yet been identified. This report identified vitamin B derivatives, including B1, B2, B3, and B6, as components of DTT water extract. Further experiments revealed that vitamin B1 stabilizes, but does not promote, the myogenesis process in C2C12 cells (Figure 7). Seon et al. reported a correlation between insufficient vitamin B12 intake and sarcopenia in older adults [69]. Fuyuko et al. indicated that vitamin B1 or B12 intake was inversely correlated with muscle loss in older people with type 2 diabetes [70]. Takumi et al. demonstrated that vitamin B6-deficient mice had fewer quiescent satellite cells, which were required for de novo myogenesis [71]. The present study is the first to report a novel role for vitamin B1 in protecting C2C12 myotubes against IL-6-induced damage by attenuating the expression of Murf-1, a muscle-specific F-box protein [72] responsible for proteolysis and muscle atrophy (Figure 7). However, the treatment with vitamin B1 alone did not significantly promote C2C12 cell differentiation, which is inconsistent with the ability of DTT water extract to induce C2C12 cell differentiation. This suggests that some other ingredients in DTT water extract could be involved in the myogenesis-promoting process. Our findings provide additional molecular evidence to support previous reports on the relationships between vitamin B1 intake and muscle wasting. Further investigation is needed to determine whether other components of DTT water extract cooperate with vitamin B1 to regulate C2C12 differentiation.

Two cadherin family members, N- and M-cadherin, were known to promote muscle regeneration; however, it was not clear which phase during muscle regeneration these cadherins were involved in [73,74]. On the other hand, β-catenin was involved in myogenesis through cooperation with Smad7/MyoD to induce transcription of myogenic genes [75]. Furthermore, M- and N-cadherin promoted myogenesis via regulating β-catenin functionality [76,77]. The precise role of E-cadherin in regulating muscle differentiation or regeneration has not been clarified. Our results showed that both E-cadherin and β-catenin were upregulated by administering DTT water extract, while decreased by IL-6 treatment in the C2C12 myotube (Figure 8B). Furthermore, we also demonstrated that upregulation of E-cadherin was required for the multinucleation of C2C12 myotubes (Figure 8C–E). Interestingly, this result was consistent with the findings that both DTT water extract and IL-6 affect the ratio of myotubes that contain more than 15 nuclei, implying a critical role of E-cadherin in promoting myotube fusion to generate myofibers. Since both DTT water extract and vitamin B1 in gradient maintained the myotube fusion, accompanied by up-regulation of E-cadherin under IL-6 treatment, it is reasonable to indicate that E-cadherin directly regulates myogenesis by promoting the myotube fusion. The LLC allograft in the C57BL/6 mice model is widely used in the preclinical study of cachexia-related cancer symptoms [78,79]. Gradually reduced body weight, muscle weight, strength, and lipid weight are reminiscent of those of cancer cachexia patients [80]. Thus, our results from the animal study can potentially refer to the human pathological subject. This study has several limitations. First, the major drawback stems from the absence of clinical trial data demonstrating that DTT water extract effectively treats muscle wasting caused by cachexia or aging. Second, the animal study employed a relatively small mouse sample size (n = 5 per group), which may impair the accuracy and validity of the findings and limit their translatability to clinical contexts. Furthermore, although vitamin B1 is present in the DTT water extract and is involved in myoblast differentiation, our results strongly suggest that compounds other than vitamin B1 in the extract are required to fully regulate this process. Additionally, the complex effects of IL-6 on myoblasts may be attributed to its concentration and the timing of its administration.

Therefore, future studies should elucidate the roles of both vitamin B1 and novel compounds within the DTT water extract in myoblast differentiation under various conditions. Ultimately, determining their clinical effectiveness against muscle atrophy resulting from cachexia or aging will be crucial.

## 4. Materials and Methods

### 4.1. DTT Extraction and HPLC Procedure

*Dendrobium* Taiseed Tosnobile (DTT, Supplementary Figure S1) was kindly provided by Dr. Chi-Luan Wen of the Taiwan Seed Improvement and Propagation Station (Taichung, Taiwan). The dried and powdered DTT stem was thoroughly mixed with pure water. The resulting mixture was shaken with an ultrasonic oscillator for approximately 60–90 min, filtered with 3M filter paper, and the filtrate was collected. In addition, the residue obtained by filtration was used to repeat the above-mentioned pure water mixing-ultrasonic vibration-filtration steps three times. Then, all the collected filtrate is concentrated under reduced pressure to remove the pure water, thereby obtaining the dendrobium water extract. The HPLC fingerprint of DTT water extract was provided by the Health Technology Center of Chung-Shan Medical University (https://rc.csmu.edu.tw/p/412-1018-23.php, accessed on 29 October 2025). Briefly, the dry matter weight of DTT (10 g) was dissolved in deionized water (1 L) and settled overnight in a refrigerator at 4 °C. The precipitate was filtered with 0.45 μm filter paper, and the filtrate was concentrated with a rotary evaporator. The net content of DTT (0.5 g) was mixed with 5 mL of sterile water in a tube. The chromatographic separation of DTT was carried out using a Shimadzu HPLC- Diode Array Detector system, equipped with an Inertsil Octadecylsilyl-4 column (250 mm × 4.6 mm, five μm). The injection volume, flow rate, and detection wavelength were 10 μL, 1.0 mL/min, and 254 nm, respectively. Separation was performed using a gradient elution of water and acetonitrile based on the following procedure: The mobile phase used for the analysis was a gradient elution system consisting of solvent A (water, 0.1% acetic acid) and solvent B (1% acetic acid in acetonitrile). The gradient program was as follows: 0–2 min, 1% solvent B; 2–22 min, 30% solvent B; 22–32 min, 70% solvent B; 32–45 min, 1% solvent B.

### 4.2. Animal Model

Adult C57BL/6 (C57 black 6) mice (8 weeks of age, 20–22 g, female) were purchased from Lasco (Taipei, Taiwan) (http://www.biolasco.com.tw/index.php/tw/ accessed on 29 October 2025), followed by housing and acclimating to their cages and human handling for 7 days before the experiments. The mice were then housed in a room maintained at 22 ± 2 °C under a 12 h light/dark cycle (5 mice per cage, lights on at 7:00 a.m.). Laboratory Rodent Diet 5001 (LabDiet, Richmond, IN, USA), which was composed of 57.5% carbohydrates, 28.9% protein, and 13.6% fat, was applied as a daily routine diet. The animal experiments were conducted by the Guide for the Care and Use of Laboratory Animals prepared by the US National Institutes of Health and procedures approved by the Institutional Animal Care and Use Committee of Chung-Shan Medical University (CSMU IACUC No. 2422, 2 March 2021) and the Animal Research: Reporting of In Vivo Experiments (ARRIVE) guideline. The LLC tumor-induced muscle-wasting model was conducted based on the previous study [81]. Briefly, 20 C57BL/6 mice were randomly assigned using a computer-based random order generator to 4 groups (5 mice per group with equal mean weights): regular, LLC, L + DWL (DTT water extract low dose, 75 mg/kg), L + DWH (DTT water extract high dose, 150 mg/kg). The mice were subcutaneously injected in the right inguinal region with 100 μL (1 × 10^5^ cells) of LLC cells, except for the standard group. Seven days after the injection of LLC cells, all mice had developed tumors, as confirmed by palpation of the injection site (approximately 0.5 cm in diameter). The tumor-bearing mice were then gavagely administered water, a low or a high dose of DTT water extract daily for 18 consecutive days until the day of sacrifice.

### 4.3. Food Intake, Body Weight, and Grip Strength

Body weight and food intake were evaluated weekly by weighing the food and the animals before gavage administration of water or DTT water extract. Both body weight and food intake were expressed in grams/week. The grip strength tests were performed using a BIO-GT3 tester (BIOSEB, Pinellas Park, FL, USA) according to the guidelines of the International Mouse Phenotyping Consortium [82]. Each mouse was placed on a metal grid connected to a horizontal force sensor. After the forelimbs of the mouse were stably grabbed on the grid, the tail was pulled backward at a 45° angle until the mouse was released from the grid, and the strength of the limb was recorded. The test for each mouse was repeated at least three times, and the results were averaged for analysis. On the day of the endpoint, all mice were sacrificed in a CO_2_ chamber. Organs (gastrocnemius and soleus, lung) and tumors were removed, weighed, and recorded.

### 4.4. Histological Analysis

The mice were sacrificed 28 days after the LLC implant for morphometric analysis, and the left hind leg was collected. The leg tissue samples were then dehydrated, embedded in paraffin, sectioned at three μm, mounted on positively charged microscope slides (Thomas Scientific, Swedesboro, NJ, USA), and dried in a 50 °C oven for 30 min. For H/E staining, indicated muscle tissue sections were stained according to the previously described and manufacturer’s protocol (https://www.leicabiosystems.com accessed on 29 October 2025). Fibers’ diameters, area, and nucleus numbers were measured in at least three randomly selected microscope fields and calculated using Fiji Image J software version 2.1.5 (https://imagej.net/software/fiji/downloads accessed on 29 October 2025). For IHC staining, the proper hind muscle tissues were fixed in 10% formalin overnight at 25 °C. The Paraffin-embedded muscle was cut into 3 µm sections using a microtome and dried for two hours at 37 °C or overnight at room temperature. The muscle sections were then deparaffinized and rehydrated using the following washing steps: 5 min xylene, three times; 5 min 100% ethanol, three times; 5 min 95% ethanol and 5 min 75% ethanol, followed by rinsing with distilled water. Next, sodium citrate buffer (10 mM; pH 6.0) was incubated with muscle slides twice for 10 min using a microwave for antigen retrieval. Muscle sections were incubated with 2% peroxidase at room temperature for 15 min, blocked with 5% BSA (*w*/*v*) at room temperature for 30 min, and incubated with anti-pan-MyHC antibody (Elabscience, Houston, TX, USA) overnight at 4 °C. The tissue sections were then incubated with a biotinylated anti-rabbit secondary antibody for one hour at room temperature. Streptavidin-horseradish peroxidase and peroxidase substrate solution were added to muscle sections at room temperature for 10 min for signal development. IHC stains were analyzed under a light microscope.

### 4.5. Cell Culture, Treatments, and Transfection

The C2C12 myoblasts (CRL-1772) were purchased from ATCC (Manassas, VA, USA). C2C12 cells were maintained in DMEM (Dulbecco’s Modified Eagle’s Medium) containing 10% FBS (Fetal Bovine Serum, Gibco standard, Mexico city, Mexico) at 37 °C with 5% CO_2_. C2C12 myoblasts were seeded in 100 mm dishes (1 × 10^6^ cells/dish) or 12-well plates (5 × 10^4^ cells/well) for myogenic differentiation. The next day, the growth medium was replaced with a differentiation medium containing 2% horse serum with or without DTT water extract. Six days later, the differentiated C2C12 myotubes were collected and subjected to the indicated assay. In the IL-6 treatment experiment, the IL-6 (100 ng/mL) was added to the differentiated C2C12 myotubes 6 days after replacing them with differentiated medium for another 48 h. It was followed by collecting and subjecting them to the indicated assay. For transfection, the scramble or mouse E-cadherin siRNA (Small Interference RNA) purchased from Santa Cruz Biotechnology (Dallas, TX, USA) was transfected into C2C12 cells with Lipofectamine 2000 reagent twice within 48 h. The scramble RNA (scRNA) or E-cadherin siRNA transfectants were subjected to myogenic differentiation for another 6 days, followed by the indicated experiments.

### 4.6. Giemsa Staining and Measurement of Myotube Diameters

The differentiated C2C12 cells were washed with 1XPBS twice and fixed in absolute methanol for 5 min, after which the myotubes were stained with Giemsa dilution buffer (containing one mM KH_2_PO_4_) for 20 min and then observed by an optical microscope (SAGE, Taipei, Taiwan). Six pictures were randomly taken from each well of the six-well plates for each condition. The diameters of three different sites in each myotube were measured using Fiji Image J software version 2.1.5. At least 200 myotubes were measured in one well, and the results were averaged for analysis.

### 4.7. Immunostaining

The differentiated C2C12 cells were fixed with 2% paraformaldehyde for 25 min before incubation with 0.05% Triton X-100 for 20 min and then blocked with 1% BSA for 60 min. Cells were probed with an anti-pan-MyHC antibody (E-AB-22021, Elabscience, Houston, TX, USA) overnight at 4 °C, followed by incubation with a TRITC or FITC-conjugated goat anti-rabbit IgG antibody (Sigma, St. Louis, MO, USA, diluted 1:200) for one hour at 37 °C, washing with PBS three times, and staining with DAPI for five minutes. The expression and location of target proteins were observed with a confocal laser scanning microscope.

### 4.8. Cytokines Analysis

Whole blood was drawn from the retroorbital sinus in experimental and control mice and collected in tubes. Blood was allowed to stay overnight at room temperature to clot, and serum (supernatant) was obtained by centrifugation at 3000 rpm at 4 °C for 5 min. The ebioscience ELISA kits (ThermoFisher, Waltham, MA, USA) were grouped to determine the levels of TNF-α and IL-6 in collected serum samples using anti-TNF-α or IL-6 antibodies, respectively. All samples were analyzed in duplicate. For the in vitro assay, the THP-1 cells (Cat. No. TIB-202, ATCC, Manassas, VA, USA) were treated with 20 ng/mL phorbol myristate acetate (Sigma, St. Louis, MO, USA) for 48 h, followed by treatment with either LPS (LipoPolySaccharide) (100 ng/mL) alone or together with DTT water extract for another eight hours. The medium was then collected and subjected to an ELISA assay kit using anti-mouse IL-6 or TNF-α antibody (ebioscience, Taipei, Taiwan), respectively.

### 4.9. Protein Extraction and Western Blot Analysis

The control or experimental cells were lysed in protein extraction solution, followed by the Bradford assay to determine protein concentrations. All samples were adjusted to an equal concentration of 50 μg/lane. The samples were then separated by 7–14% sodium dodecyl sulfate-polyacrylamide gel electrophoresis and transferred to a polyvinylidene fluoride membrane. After being blocked with 5% skim milk for three h, membranes stayed overnight at 4 °C with primary antibodies against pan-MyHC (E-AB-22021, Elabscience, TX, USA) or actin (A2228, Merck, Rahway, NJ, USA), GAPDH (sc-32233, Santa Cruz Biotechnology, Dallas, TX, USA), E-cadherin (GTX100443, GeneTex, Irvine, CA, USA), β-catenin (ab6301, Abcam, Waltham, MA, USA), respectively. The membranes were then incubated with horseradish peroxidase-conjugated goat anti-rabbit or mouse immunoglobulin G (1:4000 dilution; Santa Cruz Biotechnology, Biotechnology, Dallas, TX, USA). The blots were developed using a Chemiluminescent Substrate kit (PerkinElmer, Waltham, MA, USA).

### 4.10. Statistical Analysis

The statistical significance of differences was determined using Student’s *t*-test or ANOVA (Analysis of variance) with post hoc multiple comparison test (Tukey’s test) using Prism ver. 7.0 (GraphPad, San Diego, CA, USA). A value of *p* < 0.05 was considered significant. For all analyses, * *p* < 0.05, ** *p* < 0.01, or *** *p* < 0.001 compared to the control group.

## 5. Conclusions

Our previous findings collectively demonstrated that DTT is a non-toxic, immunomodulating, and muscle-wasting prevention herb. It is worth further investigation into other physiological benefits of DTT, and this evidence might help to elevate the medical and economic values of DTT and encourage farmers to cultivate DTT to improve public health issues.

## Data Availability

The data presented in this study are available in the article.

## Supplementary Materials

The following supporting information can be downloaded at: https://www.mdpi.com/article/10.3390/ijms262110704/s1.
