# Supplementary material for: Vitamin B1 Involved in Dendrobium Taiseed Tosnobile Extract Mediates Protection Against Cancer-Induced Muscle Wasting by Suppressing IL-6 Pathogenicity and Enhancing Myoblast Fusion
Source: Int J Mol Sci. 2025 Nov 3;26(21):10704. doi: 10.3390/ijms262110704 (PMC12609459; doi:10.3390/ijms262110704)
Supplement: Supplementary file 1 [file ijms-26-10704-s001.zip › Supplementary Figure legends.pdf]

Figure S1. Image of Dendrobium Taiseed Tosnobile.

Figure S2. The image of (A) myofibre segment or (B) abnormal nuclei aggregations, or (C) centralized nucleus indicated with a green arrow form rectus femoris (RF), gastrocnemius (GM), and soleus (SQ) muscles of different groups of mice were histologically revealed after H/E staining on the day of sacrifice. Scale bar= 50 μ m.

Figure S3. DTT water extract recovered the expression of MyHC in LLC tumor-bearing mice. After euthanasia of the mice on day 28, the hind legs were dissected and subjected to the IHC using anti-MyHC antibody. Dak brown indicated the staining region.

Figure S4. Vitamin B1 attenuates IL-6 generation of active THP-1 cells. THP-1 cells were co-treated with LPS (100 ng/ml) with different doses of vitamin B1 for eight hours, the medium was then collected and subjected to ELISA to reveal the levels of IL-6 or TNF-α , **p<0.005, *p<0.05, one-way ANOVA achieved multiple group comparisons with a Tukey's post hoc test.

Figure S5. Tumor growth curves for LLC xenografts. Mice were treated with (1) LLC group receiving no extract (red line); (2) Low dose of DTT water extract orally fed daily for four consecutive weeks (orange line); (3) Low dose of DTT water extract orally fed daily for four consecutive weeks (yellow line). The X-axis represents treatment (in days), and the Y-axis represents tumor growth difference (mm^3) from baseline tumor growth.

Figure S6. The high magnification of the histological image of the hind limb of LLC-tumor-bearing mice. After euthanasia of the mice on day 28, the histopathological analysis of RF (Rectus Femoris), GM (gastrocnemius) Sol (soleus) of mice from control, LLC, LLC plus DTT water extract low dosage, and LLC plus DTT water extract high dosage were performed using H/E stain. The 50-fold magnification of the original image, as shown in the lower-left panel, was displayed. Scale bar=50 μm

Figure S7. The percent change of fusion index, the total number of nuclei, myotube, and nuclei/myotube of differentiated C2C12 cells of different groups.
