# Supplementary figures and images for: Vitamin B1 Involved in Dendrobium Taiseed Tosnobile Extract Mediates Protection Against Cancer-Induced Muscle Wasting by Suppressing IL-6 Pathogenicity and Enhancing Myoblast Fusion
Source: Int J Mol Sci. 2025 Nov 3;26(21):10704. doi: 10.3390/ijms262110704 (PMC12609459; doi:10.3390/ijms262110704)

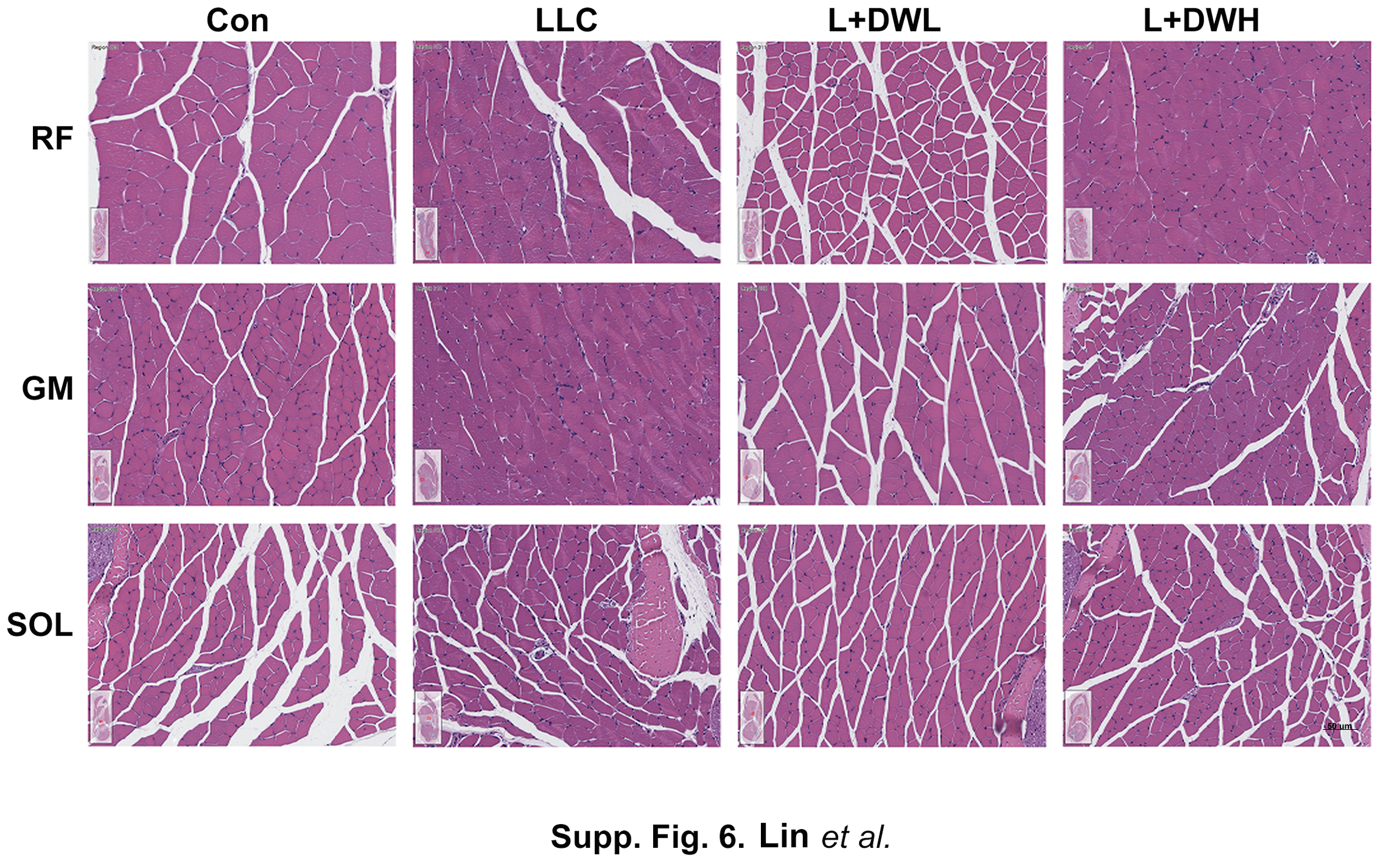

Supplement: Supplementary file 1 [file ijms-26-10704-s001.zip › supp 6.tif]

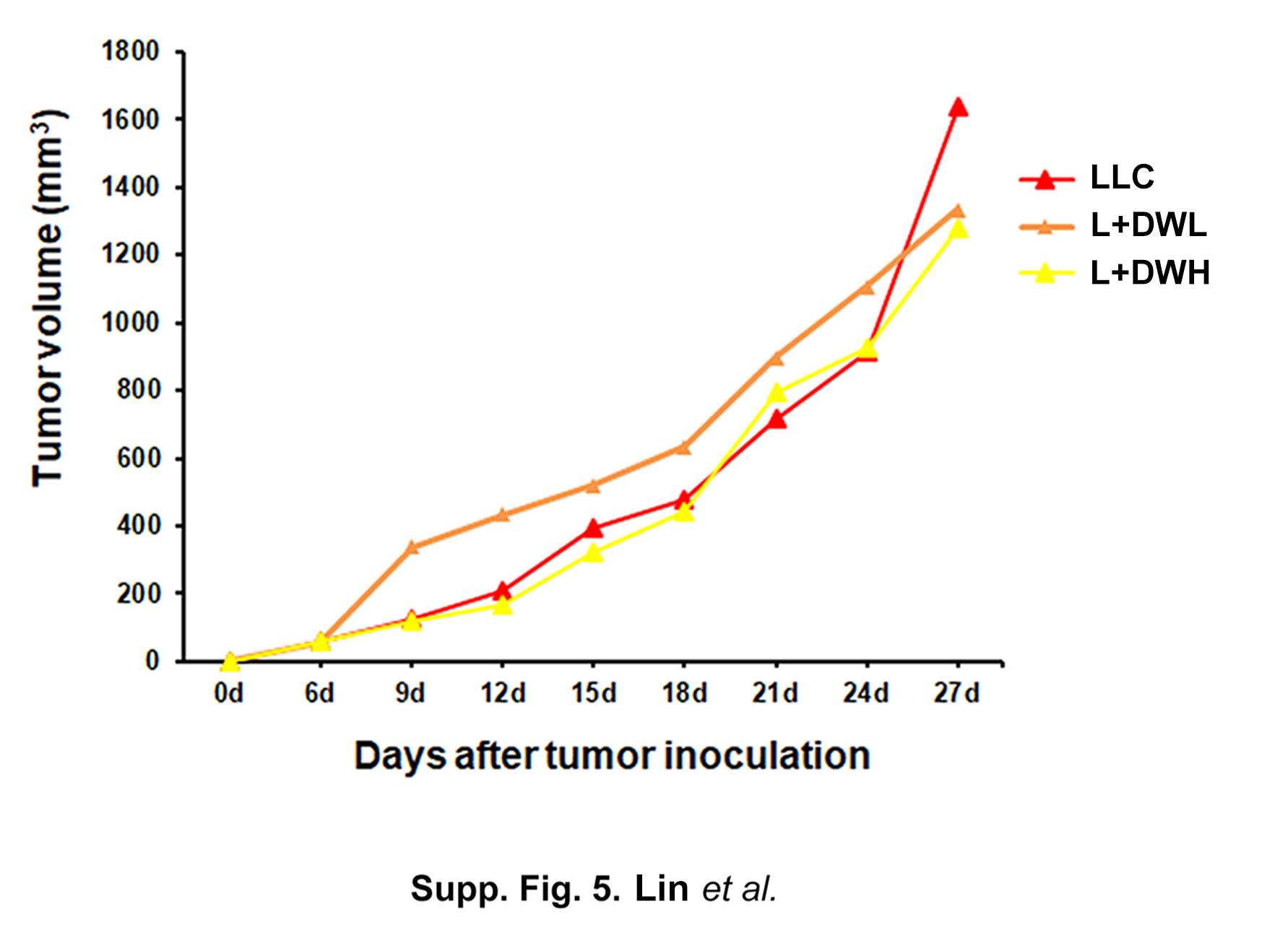

Supplement: Supplementary file 1 [file ijms-26-10704-s001.zip › supp 5.tif]

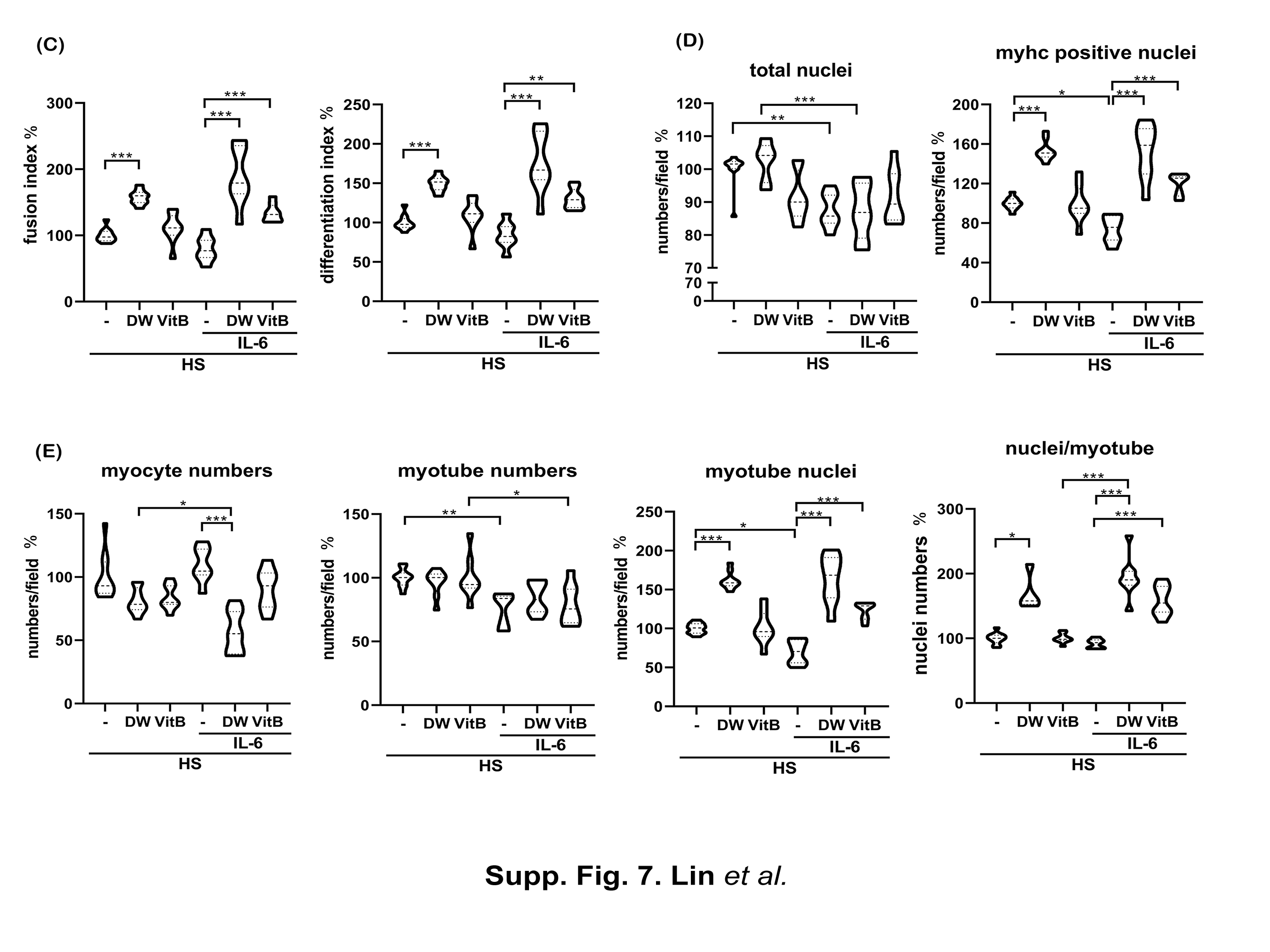

Supplement: Supplementary file 1 [file ijms-26-10704-s001.zip › supp 7.tif]

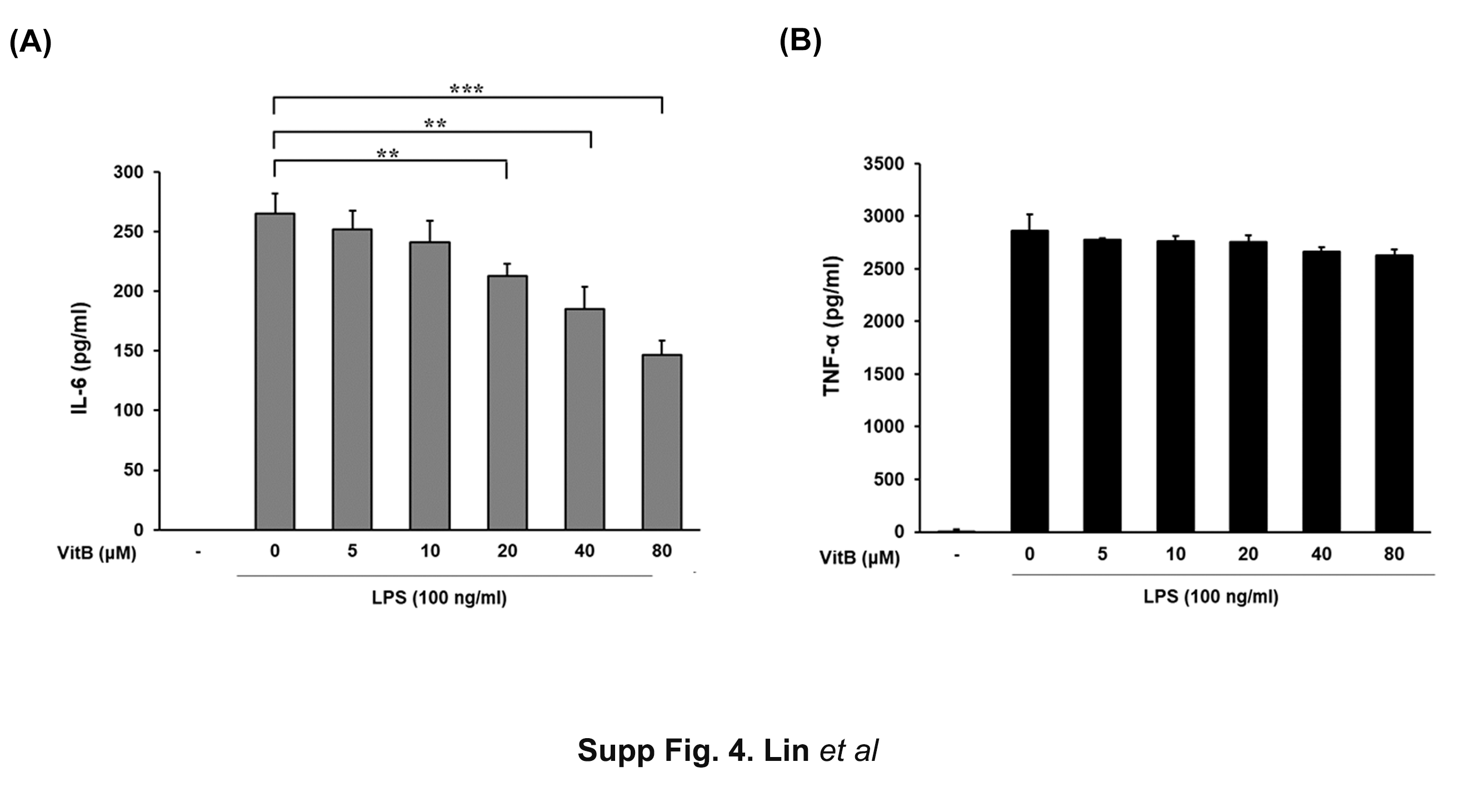

Supplement: Supplementary file 1 [file ijms-26-10704-s001.zip › supp 4.tif]

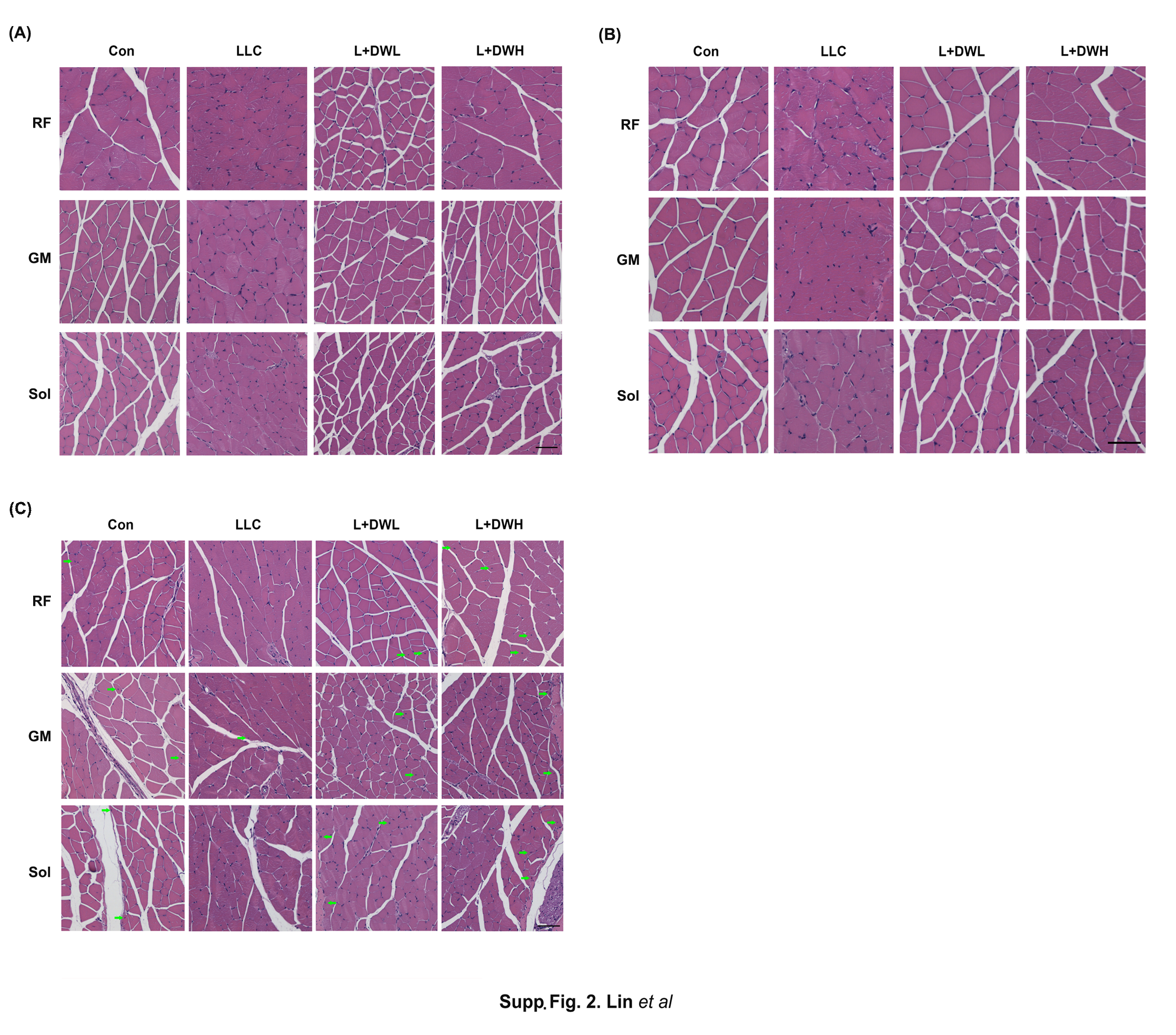

Supplement: Supplementary file 1 [file ijms-26-10704-s001.zip › supp 2.tif]

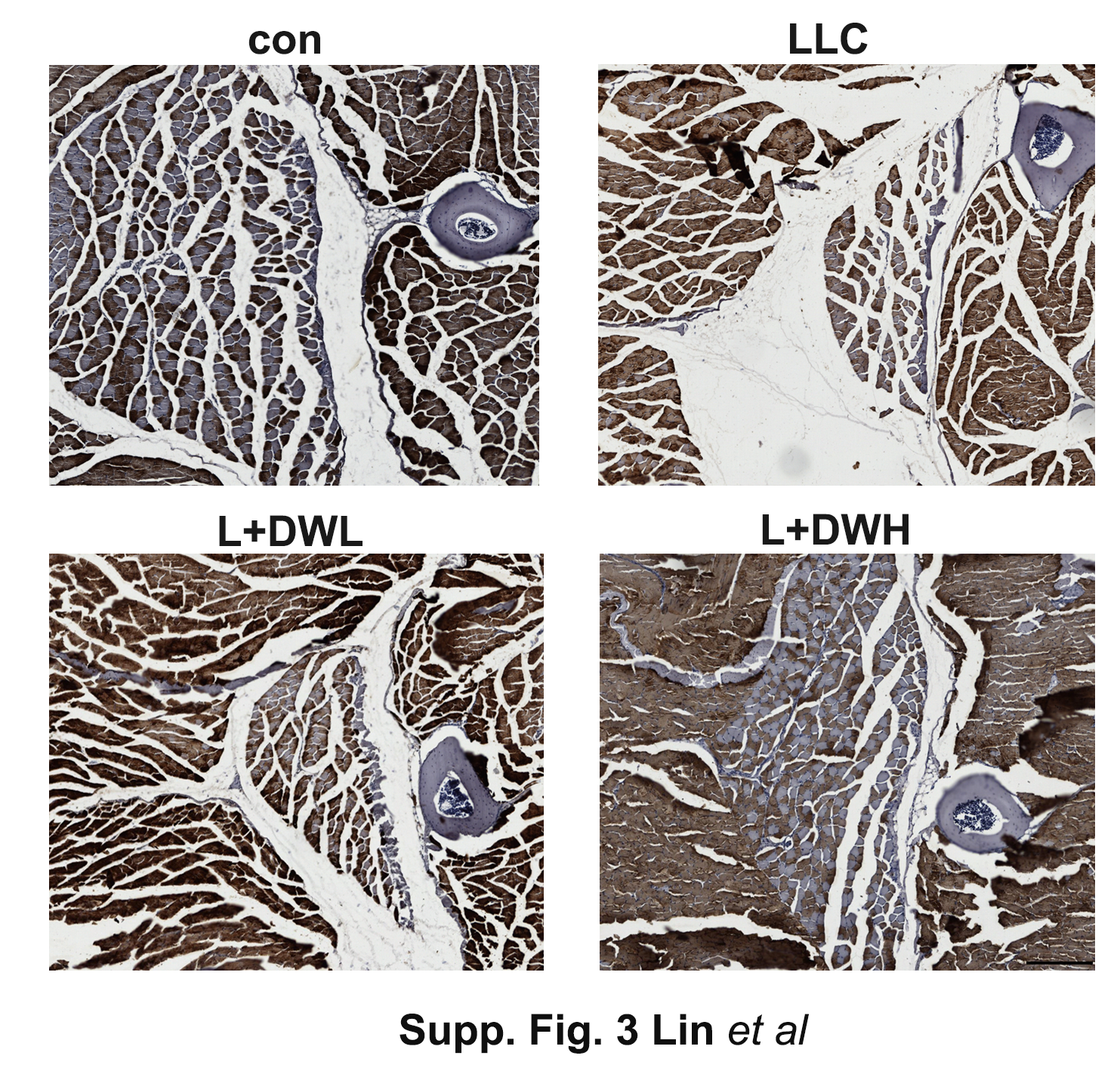

Supplement: Supplementary file 1 [file ijms-26-10704-s001.zip › supp 3.tif]

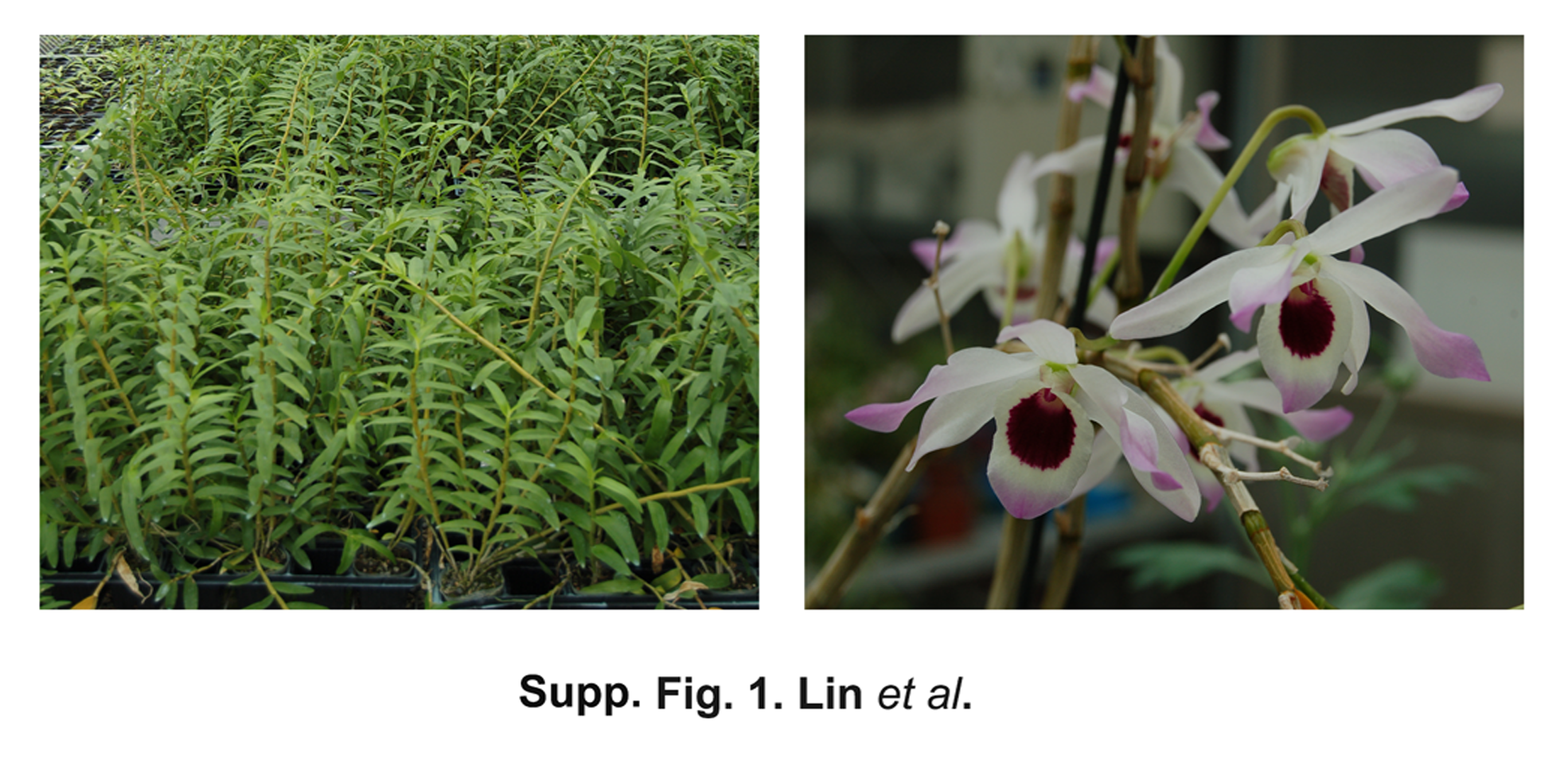

Supplement: Supplementary file 1 [file ijms-26-10704-s001.zip › supp 1.tif]
